# Graphic Groups, Graph Homomorphisms, and Graphic Group Lattices in Asymmetric Topology Cryptography

**DOI:** 10.3390/e25050720

**Published:** 2023-04-26

**Authors:** Meimei Zhao, Hongyu Wang, Bing Yao

**Affiliations:** 1 College of Science, Gansu Agricultural University, Lanzhou 730070, China; 2 National Computer Network Emergency Response Technical Team/Coordination Center of China, Beijing 100029, China; 3 College of Mathematics and Statistics, Northwest Normal University, Lanzhou 730070, China

**Keywords:** graphic group, mixed graphic group lattice, graphic coloring, graph homomorphism, graphic category, network encryption

## Abstract

Using asymmetric topology cryptography to encrypt networks on the basis of topology coding is a new topic of cryptography, which consists of two major elements, i.e., topological structures and mathematical constraints. The topological signature of asymmetric topology cryptography is stored in the computer by matrices that can produce number-based strings for application. By means of algebra, we introduce every-zero mixed graphic groups, graphic lattices, and various graph-type homomorphisms and graphic lattices based on mixed graphic groups into cloud computing technology. The whole network encryption will be realized by various graphic groups.

## 1. Introduction

### 1.1. Research Background

Cryptography is the core technology and basic support to ensure network and information security. As is well known, modern cryptography and its mathematical theories, such as lattice cryptography, are used as a kind of cryptography to resist quantum computing attacks. From Ref. [1], one can learn more about the importance and research status of lattice cryptography in the design of mathematical problems as well as its development and applications.

Xiaogang Wen, an academician of the United States, pointed out in his article entitled “New revolution in physics modern mathematics in condensed matter physics” that “But since the quantum revolution, especially, the second quantum revolution, we are more and more aware that our world is not continuous, but discrete. We should look at the world from the perspective of algebra.” Indeed, the development of modern mathematics proceeds exactly from continuous to discrete as well as from analysis to algebra. Modern mathematics also asserts the notion that discrete algebra is more essential than continuous analysis.

Group theory and, in particular, non-Abelian groups provide plenty of supply of complex and varied problems for cryptography. Over the past few decades, group-based cryptography has been extensively studied. For example, in 1999 Anshel and coauthors proposed the commutator key-exchange protocol based on the braid groups [2]. In 2004, Eick and Kahrobaei proposed the polycyclic groups as a new platform for cryptography [3]. These polycyclic groups are a natural generalization of cyclic groups with more complex algorithmic theory. In 2008, Ostrovsky and Skeith III determined sufficient and necessary conditions for the existence of a fully homomorphic encryption scheme (over a non-zero ring) if and only if homomorphic encryption exists over any finite non-Abelian simple group [4]. Since 2016, graph groups have been proposed by Flores, Kahrobaei, and Koberda for various cryptographic protocols as several of the algorithmic problems in these graph groups are NP-complete, which provides quantum-resistant cryptosystems (see, Section 7 of Ref. [5] for more detail). Moreover, in 2019 Kahrobaei and coauthors proposed the nilpotent groups for making multi-linear maps [6]. In 2021, Anshel and coauthors presented the so-called WalnutDSA™ [7], a group-based quantum-resistant public-key digital signature method on the basis of the one-way function E-multiplication. It can provide very efficient means of validating digital signatures, as the authors claimed [7], which is essential for low-powered and constrained devices. Just very recently, a complete overview of the actual state of group-based cryptography in the quantum era was updated by Kahrobaei, Flores, and Noce [8], in which some important encryption groups such as polycyclic groups and graph groups, as well as relevant combinatorial algebraic problems, are reviewed in detail.

The advantages of asymmetric encryption are as follows: higher security, the public key is public, and the private key is saved by oneself instead of sharing with others. In Ref. [9], we proposed the graphic group based on the Abelian additive operation of finite modulus in 2017, called *every-zero graphic group*. Graphic groups were further investigated in detail [10,11,12,13,14]. The mixed graphic group was introduced for the first time in Ref. [15] and then employed to encrypt networks in whole. Moreover, the infinite graphic group was also introduced [16].

Cryptographical graphs should possess the following characteristics: (1) they can be conveniently used in daily activities; (2) they are characterized by strong security, i.e., they are difficult to crack; (3) graphs and colorings (resp. labelings) are available for making topological key-pairs. In the present work, our goal is to propose some techniques of asymmetric topology cryptography for encrypting networks.

The present paper is structured as follows. After introducing basic concepts and definitions in Section 1.2, in the following section we shall focus on graphic groups by introducing mixed graphic groups and some particular mixed graphic groups such as infinite mixed graphic groups and their homomorphisms. In Section 3, some graphic lattices will be built up by several every-zero mixed graphic groups for encrypting networks. In Section 4, we will discuss the whole network encryption, such as encrypting tree-like networks.

### 1.2. Basic Concepts and Definitions

In the present paper, the terminologies and notations from Refs. [17,18,19], as well as the following notations, will be used.

Throughout this paper, let *G* be a non-trivial simple undirected graph with vertex set V(G) and edge set E(G). A graph *G* is a *(p,q)-graph* if |V(G)|=p and |E(G)|=q. A tree is a connected acyclic graph, in which a *leaf* is a vertex of degree one and any two vertices are connected by a unique path. A simple graph is called a *complete graph* if each pair of distinct vertices is joined by an edge in the graph. A complete graph of *n* vertices is denoted as $K_{n}$. A *bipartite graph H* holds V(H)=X∪Y with X∩Y=∅ such that each edge uv∈E(H) holds u∈X and v∈Y.

The cardinality of a set *X* is denoted as |X|; [a,b] indicates a set {a,a+1,a+2,⋯,b} with integers a,b holding a<b; ${\left[r,s\right]}^{o}$ denotes an odd-integer set {r,r+2,⋯,s} with odd numbers r,s holding 1≤r≤s−2 true; and $Z^{0}$ represents the set of all non-negative integers.

A graph labeling is an assignment of integers to the vertices or edges, or both, subject to certain conditions. In fact, graph labeling was first introduced in the mid 1960s, and since then approximately 200 graph-labeling techniques have been investigated [20]. In addition, the statement “a *W*-constraint proper total coloring (resp. labeling)” means one of various graph labelings, or one of various graph colorings hereafter. Graph colorings and labelings that are not defined here can be found in Refs. [20,21]. Motivated by the algebraic category, here we propose the graphic category as follows:

**Definition** **1.** *A graphic category G consists of*

*(i) A set of graphs admitting total colorings;*

*(ii) A set of morphisms from A to B for two graphs A,B∈G, which is denoted as $H_{om}\left(A,B\right)$. For two morphisms $f\in H_{om}\left(A,B\right)$ and $g\in H_{om}\left(B,C\right)$, the morphism $g\circ f\in H_{om}\left(A,C\right)$ is called composition, and it satisfies the following two axioms:*

*(1)* ***Associativity law**. For morphisms $f\in H_{om}\left(A,B\right)$, $g\in H_{om}\left(B,C\right)$, and $h\in H_{om}\left(C,D\right)$, we have (h∘g)∘f=h∘(g∘f);*

*(2)* ***Identity law**. For any morphism $f\in H_{om}\left(A,B\right)$, we have $f\circ 1_{A}=f=1_{B}\circ f$, where $1_{A}\in H_{om}\left(A,A\right)$ and $1_{B}\in H_{om}\left(B,B\right)$.*

**Definition** **2.** *A set S of graphs $S_{i}$ admitting X-constraint total colorings $f_{i}$ is called the X-constraint every-zero mixed graphic group, if there is an Abelian additive operation “$\left[{+}_{k}\right]$” on the elements of S in the following way: arbitrarily take an element $S_{k}\in S$ as the* zero*. We define the operation $S_{i}\left[{+}_{k}\right]S_{j}$ as follows:*

(1) $$S_{i}\left[{+}_{k}\right]S_{j}:=S_{i}\left[+\right]S_{j}\left[-\right]S_{k}=S_{\lambda }\in S$$

*with λ=i+j−k(mod ε) computed by*

(2) $$f_{i}\left[{+}_{k}\right]f_{j}:=\left[f_{i}\left(\omega \right)+f_{j}\left(\omega \right)-f_{k}\left(\omega \right)\right]\;\left(\mathrm{mod}\;\varepsilon \right)=f_{\lambda }\left(\omega \right)$$

*with $f_{\lambda }\left(\omega \right)\in f_{\lambda }\left(V\left(S\right)\cup E\left(S\right)\right)$ and any preappointed zero $S_{k}\in S$.*

**Definition** **3** (See also Ref. [22]). *Suppose that a (p,q)-graph G admits a W-constraint total coloring f:V(G)∪E(G)→[a,b]; a colored Topcode-matrix $T_{code}\left(G,f\right)$ of the graph G is defined as*

(3) $$\begin{array}{c} T_{code}\left(G,f\right)={\left(\begin{array}{cccc} f(x_{1}) & f(x_{2}) & \cdots & f(x_{q})\\ f(x_{1}y_{1}) & f(x_{2}y_{2}) & \cdots & f(x_{q}y_{q})\\ f(y_{1}) & f(y_{2}) & \cdots & f(y_{q}) \end{array}\right)}_{3\times q}=\left(\begin{array}{c} X_{f}\\ E_{f}\\ Y_{f} \end{array}\right)={\left(X_{f},E_{f},Y_{f}\right)}^{T} \end{array}$$

*holding the W-constraint $W\langle f\left(x_{i}\right),f\left(x_{i}y_{i}\right),f\left(y_{i}\right)\rangle =0$ for i∈[1,q]. Moreover, if G is a bipartite graph with the vertex set $V\left(G\right)=X^{v}\cup Y^{v}$ and $X^{v}\cap Y^{v}=\emptyset$, we stipulate $x_{i}\in X^{v}$ and $y_{i}\in Y^{v}$ such that $X_{f}\cap Y_{f}=\emptyset$ in Equation (3), where “W-constraint” is a mathematical constraint, or a group of mathematical constraints.*

## 2. Graphic Groups

### 2.1. Mixed Graphic Groups

Wang et al. have defined the *mixed graphic group* [15]; here, we present an improved definition of the mixed graphic group as follows:

**Definition** **4.** *Suppose that a (p,q)-graph G admits a W-constraint proper total coloring f:V(G)∪E(G)→[1,M], such that two color sets f(V(G))={f(x):x∈V(G)} and f(E(G))={f(uv):uv∈E(G)} hold a collection of restrictions. We define a colored graph set $M_{f}\left(G\right)=\left\{G_{s,k}:s\in \left[1,p\right],k\in \left[1,q\right]\right\}$ with $G_{s,k}≅G$, we define a W-constraint proper total coloring $g_{s,k}\left(x\right)=f\left(x\right)+s\;\left(\;\mathrm{mod}\;\;p\right)$ for every vertex $x\in V(G_{s,k})$, and $g_{s,k}\left(uv\right)=f\left(uv\right)+k\;\left(\;\mathrm{mod}\;\;q\right)$ for each edge $uv\in E(G_{s,k})$.*

**Lemma** **1.** *Each colored graph set $M_{f}\left(G\right)$ defined in Definition 4 forms an every-zero mixed graphic group based on the Abelian additive operation defined in Definition 2.*

**Proof.**  By Definitions 2 and 4, we define the Abelian additive operation “$G_{s,k}\left[{+}_{a,b}\right]G_{i,j}$” on the colored graph set $M_{f}\left(G\right)$ under a preappointed zero $G_{a,b}\in M_{f}\left(G\right)$ as follows,

(4) $$\left[g_{s,k}\left(w\right)+g_{i,j}\left(w\right)-g_{a,b}\left(w\right)\right]\;\left(\mathrm{mod}\;\varepsilon \right)=g_{\lambda ,\mu }\left(w\right)\in M_{f}\left(G\right)$$

for each element w∈V(G)∪E(G), where λ=s+i−a(mod p) and μ=k+j−b(mod q). As w=x∈V(G), we have ε=p, and thus Equation (4) is equivalent to

(5) $$\left[g_{s,k}\left(x\right)+g_{i,j}\left(x\right)-g_{a,b}\left(x\right)\right]\;\left(\mathrm{mod}\;p\right)=g_{\lambda ,\mu }\left(x\right)\in M_{f}\left(G\right).$$

As w=uv∈E(G), we have ε=q, and Equation (4) is also equivalent to

(6) $$\left[g_{s,k}\left(uv\right)+g_{i,j}\left(uv\right)-g_{a,b}\left(uv\right)\right]\;\left(\mathrm{mod}\;q\right)=g_{\lambda ,\mu }\left(uv\right)\in M_{f}\left(G\right).$$

Especially, as s=i=a=α, we have mod ε=mod q in Equation (4), and thus we obtain

(7) $$\left[g_{\alpha ,k}\left(uv\right)+g_{\alpha ,j}\left(uv\right)-g_{\alpha ,b}\left(uv\right)\right]\;\left(\mathrm{mod}\;q\right)=g_{\alpha ,\mu }\left(uv\right)\in M_{f}\left(G\right)$$

for uv∈E(G). When k=j=b=β, and mod ε=mod p in Equation (4), we have

(8) $$\left[g_{s,\beta }\left(x\right)+g_{i,\beta }\left(x\right)-g_{a,\beta }\left(x\right)\right]\;\left(\mathrm{mod}\;p\right)=g_{\lambda ,\beta }\left(x\right)\in M_{f}\left(G\right)$$

for x∈V(G).

We show the following facts on the colored graph set $M_{f}\left(G\right)$:

(i) *Zero.* Each graph $G_{a,b}\in M_{f}\left(G\right)$ can be determined as *zero* such that $G_{s,k}\left[{+}_{a,b}\right]G_{a,b}=G_{s,k}$.

(ii) *Uniqueness.* For $G_{s,k}\left[{+}_{a,b}\right]G_{i,j}=G_{c,d}\in M_{f}\left(G\right)$ and $G_{s,k}\left[{+}_{a,b}\right]G_{i,j}=G_{r,t}\in M_{f}\left(G\right)$, we have the facts c=s+i−a(mod p)=r and d=k+j−b(mod q)=t under the zero $G_{a,b}$.

(iii) *Inverse.* Each graph $G_{s,k}\in M_{f}\left(G\right)$ has its own *inverse*$G_{s{\;}^{\prime },k{\;}^{\prime }}\in M_{f}\left(G\right)$ holding $G_{s,k}\left[{+}_{a,b}\right]G_{s{\;}^{\prime },k{\;}^{\prime }}$$=G_{a,b}$ determined by $\left[g_{s,k}\left(w\right)+g_{s{\;}^{\prime },k{\;}^{\prime }}\left(w\right)\right]\;\left(\;\mathrm{mod}\;\;\varepsilon \right)=2g_{a,b}\left(w\right)$ for each element w∈V(G)∪E(G).

(iv) *Associative law.* Under the zero $G_{a,b}$, each triple $G_{s,k},G_{i,j},G_{c,d}\in M_{f}\left(G\right)$ holds

$$G_{s,k}\left[{+}_{a,b}\right]\left(G_{i,j}\left[{+}_{a,b}\right]G_{c,d}\right)=\left(G_{s,k}\left[{+}_{a,b}\right]G_{i,j}\right)\left[{+}_{a,b}\right]G_{c,d}$$

(v) *Commutative law.* Each pair of $G_{s,k},G_{i,j}\in M_{f}\left(G\right)$ holds $G_{s,k}\left[{+}_{a,b}\right]G_{i,j}=G_{i,j}\left[{+}_{a,b}\right]G_{s,k}$ under the zero $G_{a,b}$.

The proof of the lemma is complete. □

**Remark** **1.** *Regarding the proof of Lemma 1, there are*

*(i) By Equations (5) and (6) shown in the proof of Lemma 1, we have*

(9) $$\left[f(x)+s+f(x)+i-(f(x)+a)\right]\;\left(\mathrm{mod}\;p\right)=f\left(x\right)+s+i-a\;\left(\mathrm{mod}\;p\right)=g_{\lambda ,\mu }\left(x\right)$$

*with λ=s+i−a(mod p), and*

(10) $$\left[f(uv)+k+f(uv)+j-(f(uv)+b)\right]\;\left(\mathrm{mod}\;q\right)=f\left(uv\right)+k+j-b\;\left(\mathrm{mod}\;q\right)=g_{\lambda ,\mu }\left(uv\right)$$

*with μ=k+j−b(mod q). Thus, we obtain a formula*

(11) $$G_{s,k}\left[+\right]G_{i,j}\left[-\right]G_{a,b}=G_{\lambda ,\mu }\in M_{f}\left(G\right).$$

*(ii) We call the mixed graphic group $M_{f}\left(G\right)=\left\{G_{s,k}:s\in \left[1,p\right],k\in \left[1,q\right]\right\}$ every-zero mixed graphic group based on the Abelian additive operation “$G_{i,j}\left[{+}_{a,b}\right]G_{s,k}$” defined in Equation (4), denote it as $G=\{M_{f}\left(G\right);\left[+\right]\}$, and we present its matrix expression as follows:*

(12) $$\begin{array}{c} G={\left(\begin{array}{cccc} G_{1,1} & G_{1,2} & \cdots & G_{1,q}\\ G_{2,1} & G_{2,2} & \cdots & G_{2,q}\\ \cdots & \cdots & \cdots & \cdots \\ G_{p,1} & G_{p,2} & \cdots & G_{p,q} \end{array}\right)}_{p\times q} \end{array}$$

*(iii) The every-zero mixed graphic group G contains pq graphs in total. There are two particular every-zero graphic subgroups, $\left\{F_{v}\left(G\right);\left[+\right]\right\}=\left\{G_{s,1}:s\in \left[1,p\right]\right\}\subset G$ and $\left\{F_{e}\left(G\right);\left[+\right]\right\}=\left\{G_{1,k}:k\in \left[1,q\right]\right\}\subset G$, based on the Abelian additive operation. In fact, G contains at least (p+q) every-zero graphic subgroups.*

**Figure 1:**
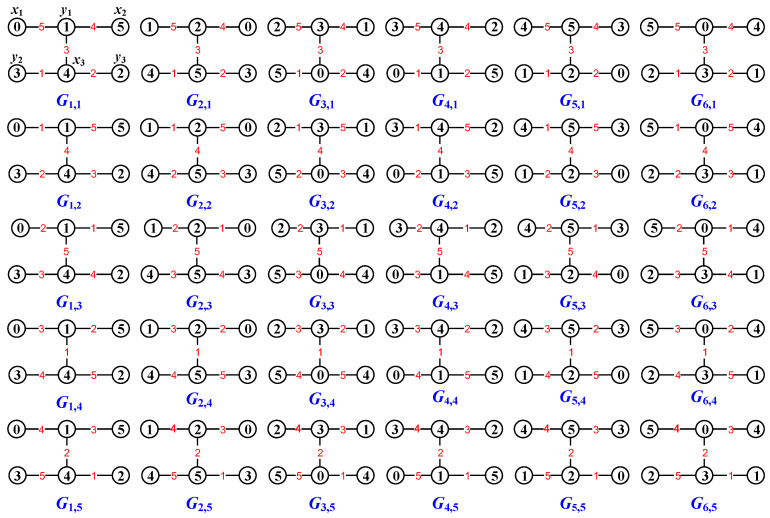
An every-zero mixed graphic group G for illustrating Definition 4 and Lemma 1.

Figure 1 shows an every-zero mixed graphic group based on a colored graph set $M_{f}\left(G\right)=\left\{G_{s,k}:s\in \left[1,6\right],k\in \left[1,5\right]\right\}$, where 6=0(mod6) and 5=5(mod5) for vertex colors, whereas 5=0(mod5) for edge colors. By using the colored graphs shown in Figure 1, one can readily verify Equation (11): $G_{s,k}\left[+\right]G_{i,j}\left[-\right]G_{a,b}=G_{\lambda ,\mu }$ for vertices and edges.

**Theorem** **1.** *Each every-zero mixed graphic group $G=\{M_{f}\left(G\right);\left[+\right]\}$ defined in Remark 1; Definitions 3 and 4 form a graphic category based on a preappointed zero $G_{a,b}\in G$ defined in Definition 1.*

**Proof.**  We define a *graphic morphism* $\theta_{a,b}\left(G_{s,k},G_{i,j}\right)$ from $G_{s,k}$ to $G_{i,j}$ by the Abelian additive operation $G_{s,k}\left[{+}_{a,b}\right]G_{i,j}$ based on a preappointed zero $G_{a,b}\in G=\left\{M_{f}\left(G\right);\left[+\right]\right\}$, that is, $\theta_{a,b}\left(G_{s,k},G_{i,j}\right):=G_{s,k}\left[{+}_{a,b}\right]G_{i,j}$. Notice that $G_{s,k}\left[{+}_{a,b}\right]G_{i,j}=G_{i,j}\left[{+}_{a,b}\right]G_{s,k}$, so $\theta_{a,b}\left(G_{s,k},G_{i,j}\right)=\theta_{a,b}\left(G_{i,j},G_{s,k}\right)$.

For $G_{i,j},G_{i+1,j+1},G_{i+2,j+2}\in G$, we define the composition of two graphic morphisms as follows:

(13) $$\begin{array}{cc} \theta_{a,b}\left(G_{i,j},G_{i,j+2}\right) & =\theta_{a,b}\left(G_{i,j},G_{i,j+1}\right)\circ \theta_{a,b}\left(G_{i,j+1},G_{i,j+2}\right)\\ \; & =\left(G_{i,j}\left[{+}_{a,b}\right]G_{i,j+1}\right)\circ \left(G_{i,j+1}\left[{+}_{a,b}\right]G_{i,j+2}\right)\\ \; & =G_{i,j}\left[{+}_{a,b}\right]G_{i,j+2} \end{array}$$

and

(14) $$\begin{array}{cc} \theta_{a,b}\left(G_{i,j},G_{i+2,j}\right) & =\theta_{a,b}\left(G_{i,j},G_{i+1,j}\right)\circ \theta_{a,b}\left(G_{i+1,j},G_{i+2,j}\right)\\ \; & =\left(G_{i,j}\left[{+}_{a,b}\right]G_{i+1,j}\right)\circ \left(G_{i+1,j}\left[{+}_{a,b}\right]G_{i+2,j}\right)\\ \; & =G_{i,j}\left[{+}_{a,b}\right]G_{i+2,j}. \end{array}$$

So, we have

(15) $$\begin{array}{cc} \theta_{a,b}\left(G_{i,j},G_{i+2,j+2}\right) & =\theta_{a,b}\left(G_{i,j},G_{i+1,j+1}\right)\circ \theta_{a,b}\left(G_{i+1,j+1},G_{i+2,j+2}\right)\\ \; & =\left(G_{i,j}\left[{+}_{a,b}\right]G_{i+1,j+1}\right)\circ \left(G_{i+1,j+1}\left[{+}_{a,b}\right]G_{i+2,j+2}\right)\\ \; & =G_{i,j}\left[{+}_{a,b}\right]G_{i+2,j+2}. \end{array}$$

Since $\theta_{a,b}\left(G_{s,k},G_{i,j}\right)\circ 1_{s,k}=\theta_{a,b}\left(G_{s,k},G_{i,j}\right)$ for $1_{s,k}=\theta_{a,b}\left(G_{s,k},G_{s,k}\right)$ and $1_{i,j}\circ \theta_{a,b}\left(G_{s,k},G_{i,j}\right)$

$=\theta_{a,b}\left(G_{s,k},G_{i,j}\right)$ for $1_{i,j}=\theta_{a,b}\left(G_{i,j},G_{i,j}\right)$, the identity law in Definition 1 holds true. The associativity law stands for graphic morphisms.

In general, by using Equations (13) and (14) repeatedly, we can obtain a *graphic morphism composition* as follows:

(16) $$\begin{array}{cc} \theta_{a,b}\left(G_{i,j},G_{s,k}\right) & =\theta_{a,b}\left(G_{i,j},G_{c,d}\right)\circ \theta_{a,b}\left(G_{c,d},G_{s,k}\right)\\ \; & =\left(G_{i,j}\left[{+}_{a,b}\right]G_{c,d}\right)\circ \left(G_{c,d}\left[{+}_{a,b}\right]G_{s,k}\right)\\ \; & =G_{i,j}\left[{+}_{a,b}\right]G_{s,k} \end{array}$$

and the *graphic morphism triangular law*.

We claim that the every-zero mixed graphic group $G=\{M_{f}\left(G\right);\left[+\right]\}$ forms a graphic category based on the graphic morphism set $H_{om}^{a,b}\left(G_{i,j},G_{s,k}\right)=\left\{\theta_{a,b}\left(G_{i,j},G_{s,k}\right):\;G_{i,j},G_{s,k}\in G\right\}$ for the preappointed zero $G_{a,b}\in G$. □

**Theorem** **2.** *Each every-zero mixed graphic group $G=\{M_{f}\left(G\right);\left[+\right]\}$ defined in Definitions 3 and 4 forms m graphic categories such as $H_{om}^{a,b}\left(G_{i,j},G_{s,k}\right)$, shown in the proof of Theorem 1, for each $G_{a,b}\in G$, where m is the number of elements of the every-zero mixed graphic group G.*

**Theorem** **3.** *A Topcode-matrix group $\left\{T_{code}\left(G_{s,k},g_{s,k}\right):G_{s,k}\in G=\left\{M_{f}\left(G\right);\left[+\right]\right\}\right\}$ based on an every-zero mixed graphic group $G=\{M_{f}\left(G\right);\left[+\right]\}$ defined in Definitions 3 and 4 forms a* Topcode-matrix category *defined in Definitions 1 and 3.*

**Remark** **2.** *(i) We take three Topcode-matrices*

$$T_{code}\left(G_{1,2},g_{1,2}\right),\;T_{code}\left(G_{3,3},g_{3,3}\right),\;T_{code}\left(G_{6,4},g_{6,4}\right)\in M_{T}=\left\{T_{code}\left(G_{s,k},g_{s,k}\right):G_{s,k}\in \left\{M_{f}\left(G\right);\left[+\right]\right\}\right\},$$

*where the Topcode-matrix set $M_{T}$ is made by the Topcode-matrices of the colored graphs of the every-zero mixed graphic group $M_{f}\left(G\right)=\left\{G_{s,k}:s\in \left[1,6\right],k\in \left[1,5\right]\right\}$ shown in Figure 1. Let $T_{code}\left(G_{6,4},g_{6,4}\right)$ be zero; we compute*

(17) $$\begin{array}{cc} \; & T_{code}\left(G_{1,2},g_{1,2}\right)\;\left[+\right]\;T_{code}\left(G_{3,3},g_{3,3}\right)\;\left[-\right]\;T_{code}\left(G_{6,4},g_{6,4}\right)\\ = & \left(\begin{array}{ccccc} 0 & 1 & 1 & 3 & 4\\ 1 & 5 & 4 & 2 & 3\\ 1 & 5 & 4 & 4 & 2 \end{array}\right)\left[+\right]\left(\begin{array}{ccccc} 2 & 3 & 3 & 5 & 0\\ 2 & 1 & 5 & 3 & 4\\ 3 & 1 & 0 & 0 & 4 \end{array}\right)\left[-\right]\left(\begin{array}{ccccc} 5 & 0 & 0 & 2 & 3\\ 3 & 2 & 1 & 4 & 5\\ 0 & 4 & 3 & 3 & 1 \end{array}\right)\\ = & \left(\begin{array}{ccccc} 3 & 4 & 4 & 0 & 1\\ 5 & 4 & 3 & 1 & 2\\ 4 & 2 & 1 & 1 & 5 \end{array}\right)=T_{code}\left(G_{4,1},g_{4,1}\right)\\ = & \left(\begin{array}{ccccc} g_{4,1}\left(x_{1}\right) & g_{4,1}\left(y_{1}\right) & g_{4,1}\left(y_{1}\right) & g_{4,1}\left(y_{2}\right) & g_{4,1}\left(x_{3}\right)\\ g_{4,1}\left(x_{1}y_{1}\right) & g_{4,1}\left(x_{2}y_{1}\right) & g_{4,1}\left(x_{3}y_{1}\right) & g_{4,1}\left(x_{3}y_{2}\right) & g_{4,1}\left(x_{3}y_{3}\right)\\ g_{4,1}\left(y_{1}\right) & g_{4,1}\left(x_{2}\right) & g_{4,1}\left(x_{3}\right) & g_{4,1}\left(x_{3}\right) & g_{4,1}\left(y_{3}\right) \end{array}\right) \end{array}$$

*under the edge modular mod 5 and the vertex modular mod6. By using the Abelian additive operation “$T_{code}\left(G_{s,k},g_{s,k}\right)\left[{+}_{a,b}\right]T_{code}\left(G_{i,j},g_{i,j}\right)$”, it is not hard to verify the Topcode-matrix set $M_{T}$ forms a Topcode-matrix group.*

*(ii) From Definition 3, each Topcode-matrix $T_{code}\left(G_{s,k},g_{s,k}\right)$ generates (3q)! number-based strings for real application. As can be seen from Equation (17), the Topcode-matrix $T_{code}\left(G_{4,1},g_{4,1}\right)$ can induce the following number-based strings:*

344015431242115,354244432110125,343124421150154

*for encrypting digital files of information networks.*

**Figure 2:**
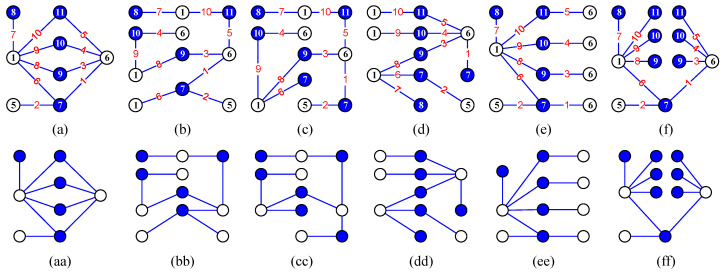
(**a**–**f**) correspond to one Topcode-matrix, but (**aa**–**ff**) are mutually not isomorphic from each other.

*(iii) Notice that a Topcode-matrix $T_{code}\left(G_{s,k},g_{s,k}\right)$ corresponds to two or more graphs, which are mutually not isomorphic from each other in general; see Figure 2 for examples. Coloring a connected graph with the elements of a Topcode-matrix group $\left\{T_{code}\left(G_{s,k},g_{s,k}\right):G_{s,k}\in G\right\}$ is a new topic in the Topcode-matrix category.*

**Theorem** **4.** *For two every-zero mixed graphic groups $\left\{M_{f}\left(G\right);\left[+\right]\right\}$ and $\left\{F_{h}\left(H\right);\left[+\right]\right\}$ defined in Remark 1, suppose that $M_{f}\left(G\right)=\left\{G_{1},G_{2},\ldots ,G_{n}\right\}$ and $F_{h}\left(H\right)=\left\{H_{1},H_{2},\ldots ,H_{n}\right\}$, and there are graph homomorphisms $G_{i}\to H_{i}$ defined by $\theta_{i}:V\left(G_{i}\right)\to V\left(H_{i}\right)$ such that each edge $uv\in E(G_{i})$ corresponds to an edge $\theta_{i}\left(u\right)\theta_{i}\left(v\right)\in E\left(H_{i}\right)$ for i∈[1,n]. Then, we obtain an every-zero mixed graphic group homomorphism,*

(18) $$\left\{M_{f}\left(G\right);\left[+\right]\right\}\to \left\{F_{h}\left(H\right);\left[+\right]\right\}.$$

### 2.2. Some Mixed Graphic Groups

#### 2.2.1. Twin Mixed Graphic Groups

**Figure 3:**
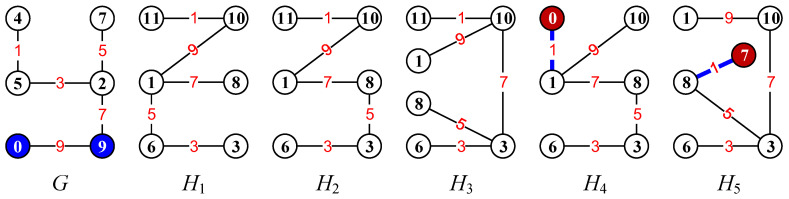
The graph *G* admits an odd-graceful labeling, which forms a twin odd-graceful matching together with each of the graphs $H_{i}$ with i∈[1,5].

In Ref. [23], the authors introduced several matching colorings (resp. labelings) of graphs and also pointed out matching diversity: configuration matching partition, coloring matching partition, set matching partition, matching chain, one-vs.-more and more-vs.-more styles of matching partitions, configuration-vs.-configuration, configuration-vs.-labeling, labeling-vs.-labeling and (configuration, labeling)-vs.-(configuration, labeling), etc. Moreover, Wang et al. [15,24] introduced the *twin odd-graceful labelings*: Suppose f:V(G)→[0,2q−1] is an odd-graceful labeling of a (p,q)-graph *G* with *p* vertices and *q* edges, and g:V(H)→[1,2q] is a labeling of another graph *H* with $p{\;}^{\prime }$ vertices and $q{\;}^{\prime }$ edges such that each edge uv∈E(H) has its own color defined as g(uv)=|g(u)−g(v)| and the edge color set $g\left(E\left(H\right)\right)={\left[1,2q-1\right]}^{o}$; we say (f,g) is a *twin odd-graceful labeling*, and *H* a *twin odd-graceful matching* of *G*. Figure 3 shows some examples of the twin odd-graceful matchings.

By the notation of Remark 1, we can obtain a *twin odd-graceful mixed graphic groups* $\left\{M_{f}\left(G\right);\left[+\right]\right\}$ and $\left\{M_{g}\left(H\right);\left[+\right]\right\}$ based on a twin odd-graceful labeling (f,g). Notice that G≇H, or G↛H, in general.

#### 2.2.2. Dual Mixed Graphic Groups

Suppose that a (p,q)-graph *G* admits a *W*-constraint total coloring f:V(G)∪E(G)→[a,b]. Let maxf=max{f(w):w∈V(G)∪E(G)} and minf=min{f(w):w∈V(G)∪E(G)}. We call the total coloring g(w)=maxf+minf−f(w) for each element w∈V(G)∪E(G)*totally dual W-constraint total coloring* of the total coloring *f*. Notice that

maxg+ming=g(w)+f(w)=maxf+minf,w∈V(G)∪E(G).

Then, $\left\{M_{g}\left(G\right);\left[+\right]\right\}$ is called a *dual mixed graphic group* of the mixed graphic group $\left\{M_{f}\left(G\right);\left[+\right]\right\}$ based on a pair of mutually dual *W*-constraint colorings *f* and *g*. Notice that these two mixed graphic groups are built up on the same graph *G*.

Respectively, we call

(i) $\alpha \left(x\right)=\max f_{v}+\min f_{v}-f\left(x\right)$ for each vertex x∈V(G) and α(uv)=f(uv) for each edge uv∈E(G) *vertex-dual W-constraint coloring* of *G*, where $\max f_{v}=\max\left\{f\left(x\right):x\in V\left(G\right)\right\}$ and $\min f_{v}=\min\left\{f\left(x\right):x\in V\left(G\right)\right\}$;

(ii) $\beta \left(uv\right)=\max f_{e}+\min f_{e}-f\left(uv\right)$ for each edge uv∈E(G) and β(x)=f(x) for each vertex x∈V(G) *edge-dual W-constraint coloring* of *G*, where $\max f_{e}=\max\left\{f\left(uv\right):uv\in E\left(G\right)\right\}$ and $\min f_{e}=\min\max\left\{f\left(uv\right):uv\in E\left(G\right)\right\}$;

(iii) (α,β) defined in (i) and (ii) *ve-separately dual W-constraint coloring* of the total coloring *f*.

**Figure 4:**
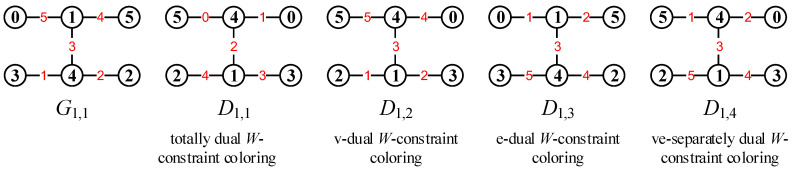
Examples for illustrating four dual colorings.

Figure 4 shows some examples for illustrating the four dual colorings mentioned above.

#### 2.2.3. Matching Mixed Graphic Groups

If a (p,q)-graph *G* is bipartite and admits a set-ordered graceful labeling *f*, there is a dozen of labelings $g_{i}$ equivalent to *f* [21,25], and thus we obtain a dozen *matching mixed graphic groups* $\left\{M_{f}\left(G\right);\left[+\right]\right\}$ and $\left\{F_{g_{i}}\left(H_{i}\right);\left[+\right]\right\}$ with i∈[1,m] for m≥2. For example, these labelings $g_{i}$ are odd-graceful labeling, odd-elegant labeling, edge-magic total labeling, image-labeling, 6C-labeling, odd-6C-labeling, even-odd separable 6C-labeling, and so on (see Ref. [23] for details). Here, we refer to the mixed graphic group $\left\{M_{f}\left(G\right);\left[+\right]\right\}$ as a *private-key*, and each mixed graphic group $\left\{F_{g_{i}}\left(H_{i}\right);\left[+\right]\right\}$ with i∈[1,m] as a *public-key* in encrypting networks.

The complement $\bar{G}$ of a simple graph *G* is the simple graph with vertex set V(G), and two vertices are adjacent in $\bar{G}$ if and only if they are not adjacent in *G*. So, we have $\left\{M_{f}\left(G\right);\left[+\right]\right\}$ and $\left\{M_{g}\left(\bar{G}\right);\left[+\right]\right\}$ as a pair of matching mixed graphic groups, where $\bar{G}$ admits a *W*-constraint coloring *g*. In general, for a graph L=G∪H with V(L)=V(G)=V(H) and E(G)∩E(H)=∅, we have $\left\{M_{f}\left(G\right);\left[+\right]\right\}$ and $\left\{F_{h}\left(H\right);\left[+\right]\right\}$ as a pair of matching mixed graphic groups based on the graph L=G∪H, where *H* admits a *W*-constraint coloring *h*.

**Figure 5:**
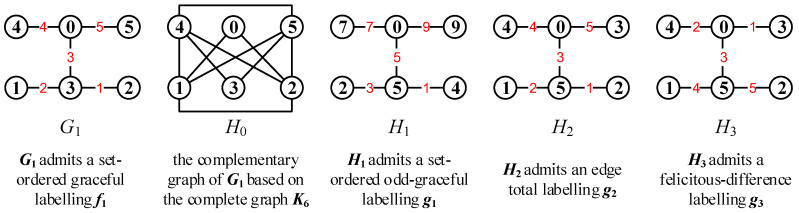
Examples of the complementary graph and three colorings $g_{1},g_{2},g_{3}$ generated from the coloring $f_{1}$ of $G_{1}$ by equivalent transformation.

Figure 5 shows the complementary graph of a given graph $G_{1}$ and some labellings generated from a given set-ordered graceful labelling $f_{1}$ of the graph $G_{1}$.

### 2.3. Infinite Mixed Graphic Groups and Their Homomorphisms

From Definition 4, we obtain an *every-zero infinite mixed graphic group*

(19) $$I_{-\infty }^{+\infty }\left(G,f;\left[+\right]\right)=\left\{G_{s,k}:-\infty <s,k<+\infty \right\}$$

with $G_{s,k}≅G$ based on a (p,q)-graph *G* admitting a *W*-constraint proper total coloring *f* and $G≅G_{0,0}$, where “[+]” is the Abelian additive operation “$G_{s,k}\left[{+}_{a,b}\right]G_{i,j}$” under a preappointed zero $G_{a,b}\in I_{-\infty }^{+\infty }\left(G,f;\left[+\right]\right)$ for any pair of graphs $G_{s,k},G_{i,j}\in I_{-\infty }^{+\infty }\left(G,f;\left[+\right]\right)$.

**Remark** **3.** *The elements of an every-zero infinite mixed graphic group $I_{-\infty }^{+\infty }\left(G,f;\left[+\right]\right)$ defined in Equation (19) can fully tile each integer point (x,y) of the xOy-plane. Moreover, $I_{-\infty }^{+\infty }\left(G,f;\left[+\right]\right)$ contains infinite every-zero mixed graphic groups having finite elements, such as $F({\left\{G_{s+i,k}\right\}}_{i=1}^{p};\left[+\right])$ and $F({\left\{G_{s,k+j}\right\}}_{j=1}^{q};\left[+\right])$. Additionally, $I_{-\infty }^{+\infty }\left(G,f;\left[+\right]\right)$ contains infinite every-zero mixed graphic groups having infinite elements.*

*$I_{-\infty }^{+\infty }\left(G,f;\left[+\right]\right)$ is also a graphic category under the graphic morphism composition defined in Equation (16). Particular every-zero mixed graphic groups having infinite elements, or finite elements can be used easily to randomly encrypt networks.*

**Theorem** **5.** *(i) Suppose that the coloring f of the (p,q)-graph G based on an every-zero infinite mixed graphic group $I_{-\infty }^{+\infty }\left(G,f;\left[+\right]\right)$ is equivalent to another $W_{g}$-constraint total coloring g of the (p,q)-graph G based on an every-zero infinite mixed graphic group $I_{-\infty }^{+\infty }\left(G,g;\left[+\right]\right)$. If a mapping φ:V(G)∪E(G)→V(G)∪E(G) exists such that g(w)=φ(f(w)) for w∈V(G)∪E(G), then we obtain an every-zero infinite mixed graphic group homomorphism,*

(20) $$I_{-\infty }^{+\infty }\left(G,f;\left[+\right]\right)\to I_{-\infty }^{+\infty }\left(G,g;\left[+\right]\right).$$

*(ii) Suppose a graph homomorphism from a (p,q)-graph G to a connected graph H based on a mapping φ:V(G)→V(H) such that each edge uv∈E(G) corresponds to an edge φ(u)φ(v)∈E(H), and vice versa. Suppose that the (p,q)-graph G admits a W-constraint total coloring f, and the graph H admits a $W{\;}^{\prime }$-constraint total coloring h. Then, we obtain an every-zero infinite mixed graphic group homomorphism as follows:*

(21) $$I_{-\infty }^{+\infty }\left(G,f;\left[+\right]\right)\to I_{-\infty }^{+\infty }\left(H,h;\left[+\right]\right).$$

*Notice that, in general, G≇H.*

## 3. Graphic Lattices

### 3.1. Mixed Graphic B-Group Lattices

**Definition** **5.** *Using an every-zero mixed graphic group $G=\{M_{f}\left(G\right);\left[+\right]\}$ defined in Remark 1 to encrypt a connected graph H by a mapping φ:V(H)∪E(H)→G such that each edge uv∈E(H) holds $\varphi \left(uv\right)=\varphi \left(u\right)\left[{+}_{a,b}\right]\varphi \left(v\right)$ under a preappointed zero $G_{a,b}\in G$, we obtain another graph L from the set {φ(x), φ(uv):x∈V(H), uv∈E(H)} by joining some vertices of the graphs $G_{i_{u},j_{u}}=\varphi \left(u\right)\in G$ and $G_{i_{v},j_{v}}=\varphi \left(v\right)\in G$ together with some vertices of the graph $G_{i_{uv},j_{uv}}=\varphi \left(uv\right)\in G$ via edges, respectively.*

**Figure 6:**
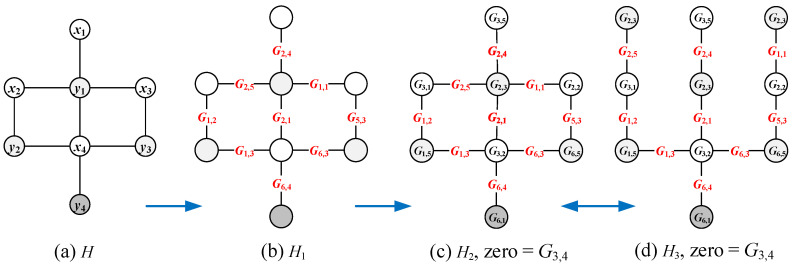
A graphic-group-coloring for illustrating Definition 5, where (**a**) is an uncolored graph *H*; (**b**) is an edge-colored graph $H_{1}$ obtained by coloring the edges of *H* with the elements of the every-zero mixed graphic group $M_{f}\left(G\right)=\left\{G_{s,k}:s\in \left[1,6\right],k\in \left[1,5\right]\right\}$ shown in Figure 1; (**c**) is a totally colored graph $H_{2}$; and (**d**) is a tree obtained from $H_{2}$ by splitting some vertices of $H_{2}$.

In Figure 6, we first use an edge coloring φ to color the edges of the uncolored graph *H* by the elements of the every-zero mixed graphic group $M_{f}\left(G\right)=\left\{G_{s,k}:s\in \left[1,6\right],k\in \left[1,5\right]\right\}$ shown in Figure 1, and then an edge-colored graph $H_{1}$ is obtained by expending this mixed graphic group edge coloring φ to the vertex set V(H), which is followed by the totally colored graph $H_{2}$. Moreover, the totally colored graph $H_{3}$ is a *colored graph homomorphism* to $H_{2}$, that is, $H_{3}\to_{color}H_{2}$.

From the proof of Lemma 1, we use the elements of an every-zero mixed graphic group $M_{f}\left(G\right)=\left\{G_{s,k}:s\in \left[1,p\right],k\in \left[1,q\right]\right\}$ based on the Abelian additive operation “$G_{i,j}\left[+\right]G_{s,k}$” defined in Equation (4) to make a *mixed graphic lattice base*, i.e.,

(22) $$\begin{array}{cc} B & =(G_{1,1},G_{2,1},\cdots ,G_{p,1},\cdots ,G_{s,k},\cdots ,G_{p,1},G_{p,2},\cdots ,G_{p,q})\\ \; & =(B_{1},B_{2},\cdots ,B_{M}), \end{array}$$

where M=pq.

**Definition** **6.** *With the notation of Equation (22), we can write the graph L in Definition 5 as $L=H{\left[{⊖}_{k}\right]}_{j=1}^{M}a_{j}B_{j}$ and call the following set:*

(23) $$\begin{array}{c} L\left(F_{m,n}\left[{⊖}_{k}\right]B\right)=\left\{H{\left[{⊖}_{k}\right]}_{j=1}^{M}a_{j}B_{j}:a_{j}\in Z^{0},B_{j}\in B,H\in F_{m,n}\right\} \end{array}$$

*under a preappointed zero $B_{k}\in B$* mixed graphic group lattice*based on a mixed graphic lattice base B, where $\sum_{j=1}^{M}a_{j}\geq 1$ and $F_{m,n}$ is a set of graphs with vertex number ≤m and edge number ≤n. Moreover, we call the following set:*

(24) $$L\left(F_{m,n}\left[⊖\right]B\right)=\left\{L\left(F_{m,n}\left[{⊖}_{k}\right]B\right):H_{k}\in B\right\}$$

mixed graphic B-group lattice*since each element of the mixed graphic lattice base B can be referred to as zero under the Abelian additive operation.*

**Remark** **4.** *Regarding Definition 5, we have*

*(i) In general, two graphs $H{\left[{⊖}_{k}\right]}_{j=1}^{M}a_{j}B_{j}$ and $H{\left[{⊖}_{s}\right]}_{j=1}^{M}a_{j}B_{j}$ are not isomorphic from each other for two different zeros $B_{k},B_{s}\in B$.*

**Figure 7:**
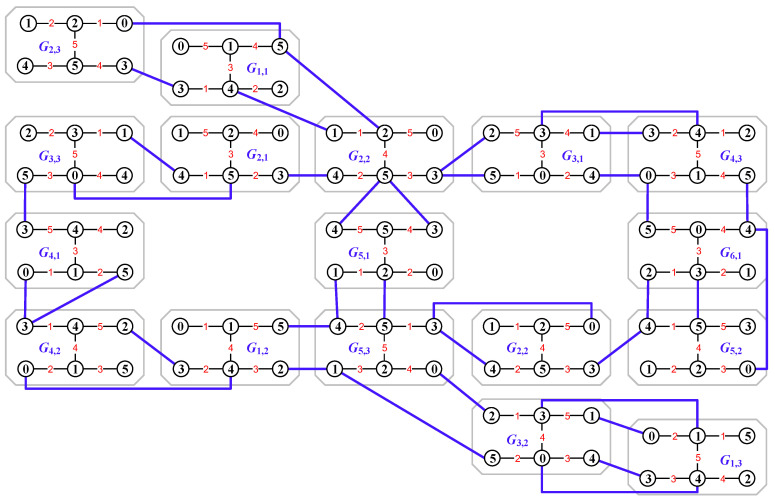
A graph *L* for illustrating Definition 6 and Remark 4 (ii). *L* is obtained from the totally colored graph $H_{2}$ shown in Figure 6 by the edge-join operation and the every-zero mixed graphic group shown in Figure 1.

*(ii) There are many different ways to join the graph $G_{i_{uv},j_{uv}}=\varphi \left(uv\right)$ with two graphs $G_{i_{u},j_{u}}=\varphi \left(u\right)$ and $G_{i_{v},j_{v}}=\varphi \left(v\right)$ by edges in Definition 5; in other words, the number of graphs of forming $H{\left[{⊖}_{k}\right]}_{j=1}^{M}a_{j}B_{j}$ is two or more, see Figure 7.*

*(iii) Since two graphs $B_{k},B_{s}\in B$ form two homomorphically equivalent graph homomorphisms $B_{k}\to B_{s}$, we obtain the following* mixed graphic group lattice homomorphisms*:*

(25) $$L\left(F_{m,n}\left[{⊖}_{k}\right]B\right)\to L\left(F_{m,n}\left[{⊖}_{s}\right]B\right)\to L\left(F_{m,n}\left[{⊖}_{k}\right]B\right).$$

*This technology has great potential for cloud computation in the future of quantum computing.*

### 3.2. Graphic Lattices Made by Graph Matchings

In the following discussion, we will use traditional complementary graphs and *G*-complementary graphs to build up graphic lattices.

#### 3.2.1. Traditional Graph and Its Complement

Let $\bar{G}$ be the *complement* of a simple graph *G*; then, we say that $\left(G,\bar{G}\right)$ is a *complete-graphic matching*. For a graph operation “(•)”, we have a *complementary mixed graphic lattice*

(26) $$L\left({\bar{F}}_{m,n}\left(•\right)B\right)=\left\{\bar{G}{\left(•\right)}_{i=1}^{M}a_{i}B_{i}:\;a_{i}\in Z^{0},\;B_{i}\in B,\;\bar{G}\in {\bar{F}}_{m,n}\right\},$$

where the mixed graphic lattice base $B=(B_{1},B_{2},\cdots ,B_{M})$ is defined in Equation (22), ${\bar{F}}_{m,n}$ is the set of all complements of graphs of $F_{m,n}$ defined in Definition 6, and $\sum_{i=1}^{M}a_{i}\geq 1$.

Let $\bar{B}=\left({\bar{B}}_{1},{\bar{B}}_{2},\cdots ,{\bar{B}}_{M}\right)$ be the *complementary base* of the mixed graphic lattice base B with the complement ${\bar{B}}_{i}$ of $B_{i}$ for i∈[1,M]. We obtain a *complementary mixed graphic lattice*

(27) $$L\left(F_{m,n}\left(•\right)\bar{B}\right)=\left\{G{\left(•\right)}_{i=1}^{M}a_{i}{\bar{B}}_{i}:\;a_{i}\in Z^{0},\;{\bar{B}}_{i}\in \bar{B},\;G\in F_{m,n}\right\}$$

with $\sum_{i=1}^{M}a_{i}\geq 1$. Moreover, we obtain a *totally complementary mixed graphic lattice* as follows:

(28) $$L\left({\bar{F}}_{m,n}\left(•\right)\bar{B}\right)=\left\{\bar{G}{\left(•\right)}_{i=1}^{M}a_{i}{\bar{B}}_{i}:\;a_{i}\in Z^{0},\;{\bar{B}}_{i}\in \bar{B},\;\bar{G}\in {\bar{F}}_{m,n}\right\}$$

with $\sum_{i=1}^{M}a_{i}\geq 1$.

We call $L(F_{m,n}\left[{⊖}_{k}\right]B)$ and $L({\bar{F}}_{m,n}\left(•\right)\bar{B})$ a matching of *complementary mixed graphic lattices*. However, for each graph $G^{*}=G{\left(•\right)}_{i=1}^{M}a_{i}B_{i}$ of $L(F_{m,n}\left[{⊖}_{k}\right]B)$, the complementary graph $\bar{G^{*}}$ of $G^{*}$ is not a graph $\bar{G}{\left(•\right)}_{i=1}^{M}a_{i}{\bar{B}}_{i}$ of $L({\bar{F}}_{m,n}\left(•\right)\bar{B})$, in general.

#### 3.2.2. *G*-Complementary

A graph *G* has two proper subgraphs $G_{1}$ and $G_{2}$ such that $V\left(G\right)=V\left(G_{1}\right)=V\left(G_{2}\right)$, $E\left(G_{1}\right)\cap E\left(G_{2}\right)=\emptyset$, and $E\left(G_{1}\right)\cup E\left(G_{2}\right)=E\left(G\right)$. Thereby, we call $\left(G_{1},G_{2}\right)$ a *G-matching*. Accordingly, we have the *G-complementary mixed graphic lattice* like that defined in Equation (28).

## 4. Encrypting Networks in Whole

In asymmetric topology cryptography, one would encrypt graphs (resp. networks) by mixed graphic groups, and we call these colorings *mixed graphic group colorings*. For the number $N_{m}$ of graphs of *n* vertices, Harary and Palmer [26] computed two graph numbers

(29) $$\begin{array}{cc} N_{23} & =559946939699792080597976380819462179812276348458981632\approx 2^{179}\\ N_{24} & =195704906302078447922174862416726256004122075267063365754368\approx 2^{197}. \end{array}$$

The large number of graphs, and of colorings in graph theory, can provide us with flexible and diverse asymmetric topology technology with stable security performance and can also increase the technical cost and intolerable time cost to the cracker. Encrypting networks in whole is an application of mixed graphic groups and mixed graphic group lattices.

### 4.1. Mixed Graphic Group Colorings in Encrypting Networks

Here, we present a proof for the following theorem, as shown partly in Ref. [16]:

**Theorem** **6.** *For each graph L of a graphic B-group lattice $L(F_{m,n}\left[⊖\right]B)$ defined in Definition 5, Equations (23) and (24) form an every-zero mixed graphic group $\left\{F_{\alpha }\left(L\right);\left[+\right]\right\}$ defined in Remark 1, where the graph L admits a total coloring α.*

**Proof.**  Suppose that a (p,q)-graph *G* admits a total coloring *f* and *L* is a graph of a graphic B-group lattice $L(F_{m,n}\left[{⊖}_{k}\right]B)$, so $L=H{\left[{⊖}_{k}\right]}_{j=1}^{M}a_{j}B_{j}$ as defined in Equation (23) and Definition 5.

Notice that each graph $G_{s,k}\in G$ defined in Remark 1 admits a *W*-constraint proper total coloring $g_{s,k}$ in Definition 4. Suppose the graph *L* admits a total coloring φ:V(L)∪E(L)→G, then each edge uv∈E(L) holds $\varphi \left(uv\right)=G_{i_{uv},j_{uv}}\in G$, $\varphi \left(u\right)=G_{i_{u},j_{u}}\in G$ and $\varphi \left(v\right)=G_{i_{v},j_{v}}\in G$, such that

(30) $$G_{i_{uv},j_{uv}}=\varphi \left(uv\right)=\varphi \left(u\right)\left[{+}_{a,b}\right]\varphi \left(v\right)=G_{i_{u},j_{u}}\left[{+}_{a,b}\right]G_{i_{v},j_{v}}$$

with $i_{uv}=i_{u}+i_{v}-a\;\left(\mathrm{mod}\;p\right)$ and $j_{uv}=j_{u}+j_{v}-b\;\left(\mathrm{mod}\;q\right)$, under a preappointed zero $G_{a,b}\in G$. In the graph *L*, there is at least one edge between $\varphi \left(u\right)=G_{i_{u},j_{u}}$ and $\varphi \left(uv\right)=G_{i_{uv},j_{uv}}$, and there is at least one edge between $\varphi \left(v\right)=G_{i_{v},j_{v}}$ and $\varphi \left(uv\right)=G_{i_{uv},j_{uv}}$.

Now, let us define a total coloring α for the graph *L* as follows:

(i) $\alpha \left(w\right)=g_{s,k}\left(w\right)$ for each element $w\in V\left(G_{s,k}\right)\cup E\left(G_{s,k}\right)\subset V\left(L\right)\cup E\left(L\right)$ if $G_{s,k}\subset L$.

(ii) For an edge xy∈E(L) holding $x\in V(G_{s,k})$ and $y\in V(G_{i,j})$, we color this edge xy with $\alpha \left(xy\right)=g_{s,k}\left(x\right)+g_{i,j}\left(y\right)\;\left(\mathrm{mod}\;q\right)$.

Next, we shall make the copies $L_{i,j}$ of the graph *L* with $L_{i,j}≅L$ for $i\in [1,p^{*}]$ and $j\in [1,q^{*}]$, where $p^{*}=\left|V\left(L\right)\right|$ and $q^{*}=\left|E\left(L\right)\right|$, and then put the copies into a set $S\left(L\right)=\{L_{i,j}:i\in \left[1,p^{*}\right],j\in \left[1,q^{*}\right]\}$. Moreover, we define a total coloring $\beta_{i,j}$ for each graph $L_{i,j}$ by setting

(i) $\beta_{i,j}\left(u\right)=\alpha \left(u\right)+i\;\left(\mathrm{mod}\;p^{*}\right)$ for each vertex $u\in V(L_{i,j})$;

(ii) $\beta_{i,j}\left(uv\right)=\alpha \left(uv\right)+j\;\left(\mathrm{mod}\;q^{*}\right)$ for each edge $uv\in E(L_{i,j})$;

(iii) For an edge xy∈E(L) holding $x\in V(G_{s,k})$ and $y\in V(G_{i,j})$, we color this edge xy with $\beta_{s,k}\left(x\right)+\beta_{i,j}\left(y\right)\;\left(\mathrm{mod}\;q^{*}\right)$.

For a preappointed zero $L_{a,b}\in S\left(L\right)$, we have the following Abelian additive operation “$L_{i,j}\left[{+}_{a,b}\right]L_{s,k}$”:

(31) $$L_{i,j}\left[{+}_{a,b}\right]L_{s,k}:=L_{i,j}\left[+\right]L_{s,k}\left[-\right]L_{a,b}=L_{\lambda ,\mu }\in S\left(L\right)$$

for any two graphs $L_{i,j},L_{s,k}\in S\left(L\right)$, such that

(32) $$\beta_{i,j}\left(w\right)+\beta_{s,k}\left(w\right)-\beta_{a,b}\left(w\right)=\beta_{\lambda ,\mu }\left(w\right)$$

holds true as $\lambda =i+s-a\;(\mathrm{mod}\;p^{*})$ and $\lambda =j+k-b\;(\mathrm{mod}\;q^{*})$.

We show that the set S(L) holds the following facts:

(i) *Zero*. Every graph $L_{a,b}\in S\left(L\right)$ can be as *zero* such that $L_{i,j}\left[{+}_{a,b}\right]L_{a,b}:=L_{i,j}$ for any graph $L_{i,j}$ of S(L).

(ii) *Closure law*. For each preappointed zero $L_{a,b}$, we have

$$L_{i,j}\left[{+}_{a,b}\right]L_{s,k}:=L_{i,j}\left[+\right]L_{s,k}\left[-\right]L_{a,b}=L_{\lambda ,\mu }\in S\left(L\right)$$

(iii) *Inverse.* Every graph $L_{i,j}\in S\left(L\right)$ has its own *inverse* $L_{i^{-1},j^{-1}}\in S\left(L\right)$ with $i^{-1}=2a-i$ and $j^{-1}=2b-j$, such that $L_{i,j}\left[{+}_{a,b}\right]L_{i^{-1},j^{-1}}:=L_{a,b}$.

(iv) *Associative law*. $\left(L_{i,j}\left[{+}_{a,b}\right]L_{s,k}\right)\left[{+}_{a,b}\right]L_{c,d}=L_{i,j}\left[{+}_{a,b}\right]\left(L_{s,k}\left[{+}_{a,b}\right]L_{c,d}\right)$.

(v) *Commutative law*. $L_{i,j}\left[{+}_{a,b}\right]L_{s,k}=L_{s,k}\left[{+}_{a,b}\right]L_{i,j}$.

Thereby, the set S(L) forms an every-zero mixed graphic group, denoted as $S\left(L\right)=\{F_{\alpha }\left(L\right);\left[+\right]\}$, and the set S(L) is a *graphic category* under the graphic morphism composition defined in Equation (16).

We can define another total coloring $\gamma_{i,j}$ for each graph $L_{i,j}\in S\left(L\right)$ by making

(i) $\gamma_{i,j}\left(x\right)=\alpha \left(x\right)+i\;\left(\mathrm{mod}\;p\right)$ for each vertex $x\in V(L_{i,j})$;

(ii) $\gamma_{i,j}\left(xy\right)=\alpha \left(xy\right)+j\;\left(\mathrm{mod}\;q\right)$ for each edge $xy\in E(L_{i,j})$;

(iii) For an edge uv∈E(L) holding $x\in V(G_{s,k})$ and $y\in V(G_{i,j})$, we color this edge uv with $\gamma_{s,k}\left(u\right)+\gamma_{i,j}\left(v\right)\;\left(\mathrm{mod}\;q\right)$, such that the set S(L) forms an every-zero mixed graphic group $S\left(L\right)=\{F_{\alpha }\left(L\right);\left[+\right]\}$.

The proof of the theorem is complete. □

### 4.2. Encrypting Tree-like Networks

As tree-like networks are easily accessible in real applications, have simple structures, and admit a lot of colorings, we will apply *mixed graphic group colorings* to encrypt tree-like networks. A tree *T* admits a mixed graphic group total coloring

$$\theta :V\left(T\right)\cup E\left(T\right)\to G=\{M_{f}\left(G\right);\left[+\right]\}$$

as defined in Remark 1, where $M_{f}\left(G\right)=\left\{G_{i,j}:i\in \left[1,p\right],j\in \left[1,q\right]\right\}$.

**Theorem** **7.** *A tree T with its maximum degree *Δ* admits a* mixed graphic group total coloring *θ from V(T)∪E(T) to a mixed graphic group $G=\{M_{f}\left(G\right);\left[+\right]\}$ defined in Remark 1 and pq>Δ, such that θ(uv)≠θ(uw) for any pair of adjacent edges uv and uw of T.*

**Proof.**  We construct another tree H=T−wz by removing a leaf *w* of the tree *T*, where the leaf *w* is adjacent to the vertex *z* of *T*, and keep the maximum degree Δ(H)=Δ(T). Assume that the tree H=T−wz admits a mixed graphic group total coloring *h* from V(H)∪E(H) to a mixed graphic group $G=\{M_{f}\left(G\right);\left[+\right]\}$ defined in Remark 1 and pq>Δ(H), such that the colors h(uv)≠h(uw) for any pair of adjacent edges uv and uw of *H*.

Let $N\left(z\right)=\left\{x_{1},x_{2},\cdots ,x_{k},w\right\}$ be the set of neighboring vertices of the vertex *z* in the tree *T*. We define a mixed graphic group total coloring $\theta :V\left(T\right)\cup E\left(T\right)\to G=\{M_{f}\left(G\right);\left[+\right]\}$ as θ(x)=h(x) for x∈V(T)∪E(T)∖{w,wz}, $\theta \left(wz\right)=G_{i_{0},j_{0}}\in G\setminus \left\{h\left(zx_{i}\right):i\in \left[1,k\right]\right\}$, and $\theta \left(w\right)=G_{s_{0},k_{0}}\in G\setminus \left\{h\left(z\right),h\left(zx_{i}\right):i\in \left[1,k\right]\right\}$, such that $\theta \left(wz\right)=h\left(z\right)\left[{+}_{a,b}\right]\theta \left(w\right)$ under a preappointed zero $G_{a,b}\in G$.

We obtain the proof of the theorem. □

**Theorem** **8.** *Each tree T of n edges admits a*mixed graphic group total coloring *θ from V(T)∪E(T) to a mixed graphic group $G=\{M_{f}\left(G\right);\left[+\right]\}$ defined in Remark 1, such that the edge index set $\{\left(i,j\right):\theta \left(u_{i}v_{j}\right)=G_{i,j}\in G,\;u_{i}v_{j}\in E\left(T\right)\}=X$, where $X=\{\left(i_{1},j_{1}\right),\left(i_{2},j_{2}\right),\cdots ,\left(i_{n},j_{n}\right)\}$ with $\left(i_{s},j_{s}\right)\neq \left(i_{k},j_{k}\right)$ for s≠k is a preappointed index set.*

**Proof.**  Assume that any tree *T* of n−1 edges holds this theorem and *T* admits a mixed graphic group total coloring F:V(T)∪E(T)→G, such that each edge uv∈E(T) is colored with

$$G_{\lambda ,\mu }=F\left(uv\right)=F\left(u\right)\left[{+}_{a,b}\right]F\left(v\right)=G_{i,j}\left[{+}_{a,b}\right]G_{s,k}$$

under a preappointed zero $G_{a,b}\in G$, and the edge index set is just

$$\left\{\left(\lambda ,\mu \right):F\left(u_{\lambda }v_{\mu }\right)=G_{\lambda ,\mu }\in G,\;u_{\lambda }v_{\mu }\in E\left(T\right)\right\}=X\setminus \left\{\left(i_{n},j_{n}\right)\right\}.$$

We add a new vertex *w* to the tree *T* by joining *w* with any vertex *x* of *T* via a new edge xw. The resulting tree is denoted as H=T+{w,xw}. Obviously, the tree *H* has *n* vertices.

We define a mixed graphic group total coloring θ:V(H)∪E(H)→G, such that each element z∈V(T)∪E(T)⊂V(H)∪E(H) is colored with θ(z)=F(z).

For the vertex *w* and the edge xw of the tree H=T+{w,xw}, we set $\theta \left(w\right)=G_{\alpha ,\beta }$ and $\theta \left(xw\right)=G_{i_{n},j_{n}}\in G\setminus \theta \left(E\left(T\right)\right)$ such that

$$G_{i_{n},j_{n}}=\theta \left(xw\right)=\theta \left(x\right)\left[{+}_{a,b}\right]\theta \left(w\right)=G_{\gamma ,\delta }\left[{+}_{a,b}\right]G_{\alpha ,\beta }$$

with $i_{n}=\gamma +\alpha -a$(mod p) and $j_{n}=\delta +\beta -b$(mod q), where the edge color set $\theta \left(E\left(T\right)\right)=\left\{G_{\lambda ,\mu }:F\left(uv\right)=G_{\lambda ,\mu }\in G,\;uv\in E\left(T\right)\right\}$. Finally, we obtain the desired edge index set

$$\{\left(i,j\right):\theta \left(x_{i}y_{j}\right)=G_{i,j}\in G,\;x_{i}y_{j}\in E\left(H\right)\}=X$$

and the theorem follows from the induction. □

**Corollary** **1.** *If a connected graph H of n edges admits a*mixed graphic group total coloring *θ from V(H)∪E(H) to a mixed graphic group $G=\{M_{f}\left(G\right);\left[+\right]\}$ defined in Remark 1, such that the edge index set $I_{ndex}=\left\{\left(i,j\right):\theta \left(u_{i}v_{j}\right)=G_{i,j}\in G,\;u_{i}v_{j}\in E\left(H\right)\right\}$, where $I_{ndex}=\left\{\left(i_{1},j_{1}\right),\left(i_{2},j_{2}\right),\cdots ,\left(i_{n},j_{n}\right)\right\}$ with $\left(i_{s},j_{s}\right)\neq \left(i_{k},j_{k}\right)$ for s≠k is a preappointed edge index set, then the connected graph H corresponds to at least a tree T of n edges such that T holds Theorem 8, and there is a* colored graph homomorphism *$T\to_{color}H$.*

**Theorem** **9.** *The edges of a tree T can be colored arbitrarily by a mixed graphic group proper edge coloring φ from the edge set E(T) to a mixed graphic group $G=\{M_{f}\left(G\right);\left[+\right]\}$ defined in Remark 1, and then this mixed graphic group proper edge coloring φ can be expended to the vertex set V(T), such that each edge uv∈E(T) holds $\varphi \left(uv\right)=\varphi \left(u\right)\left[{+}_{a,b}\right]\varphi \left(v\right)$ under a preappointed zero $G_{a,b}\in G$.*

**Proof.**  Let $G_{a,b}\in G$ be a preappointed zero. Suppose that a tree *T* of *p* vertices admits a mixed graphic group edge coloring F:E(T)→G, and this coloring *F* has been expended to V(T), such that $F\left(uv\right)=F\left(u\right)\left[{+}_{a,b}\right]F\left(v\right)$ for each edge uv∈E(T), and F(uv)≠F(uw) for any pair of adjacent edges uv,uw∈E(T). We construct a new tree H=T+{w,xw} by adding a new vertex *w* to the tree *T* and a new edge xw with x∈E(T).

For this new tree *H*, we define a mixed graphic group edge coloring φ:E(H)→G with φ(uv)=F(uv) if uv∈E(T)⊂E(H), and the mixed graphic group edge coloring φ can be expended to V(T)⊂V(H), such that $\varphi \left(uv\right)=\varphi \left(u\right)\left[{+}_{a,b}\right]\varphi \left(v\right)$ for each edge uv∈E(T) by the induction. Next, we take $\varphi \left(w\right)=G_{\alpha }\in G$ and $\varphi \left(xw\right)=G_{\lambda }\in G\setminus \left\{\varphi \left(xy_{i}\right):y_{i}\in N\left(x\right)\right\}$ holding $G_{\lambda }=\varphi \left(xw\right)=\varphi \left(x\right)\left[{+}_{a,b}\right]\varphi \left(w\right)=G_{\beta }\left[{+}_{a,b}\right]G_{\alpha }$ with λ=α+β−k(mod q). Finally, we expend the mixed graphic group proper edge coloring φ to V(H), such that $\varphi \left(xy\right)=\varphi \left(x\right)\left[{+}_{a,b}\right]\varphi \left(y\right)$ for each edge xy∈E(H), and φ(uv)≠φ(uw) for any pair of adjacent edges uv,uw∈E(H); thus, the induction is complete. □

### 4.3. Graphic Lattices for the Encryption of Dynamic Networks

For the encryption of dynamic networks, we define the following every-zero dynamically mixed graphic group: an every-zero dynamically mixed graphic group $G\left(t\right)=\{M_{f_{t}}\left(G\right);\left[+\right]\}$ is based on a dynamically colored graph set $M_{f_{t}}\left(G\right)=\left\{G_{i,j}\left(t\right):i\in \left[1,p\right],j\in \left[1,q\right]\right\}$ with $G_{s,k}\left(t\right)≅G\left(t\right)$ for t∈[α,β], where a (p,q)-graph G(t) admits a *W*-constraint proper total coloring $f_{t}:V\left(G\right)\cup E\left(G\right)\to \left[1,n\left(t\right)\right]$ for t∈[α,β], and each graph $G_{i,j}\left(t\right)$ admits a *W*-constraint proper total coloring $g_{s,k}^{t}\left(x\right)=f_{t}\left(x\right)+s\;\left(\mathrm{mod}\;p\right)$ for every vertex $x\in V(G_{i,j}\left(t\right))$, and $g_{s,k}^{t}\left(uv\right)=f_{t}\left(uv\right)+k\;\left(\mathrm{mod}\;q\right)$ for each edge $uv\in E(G_{i,j}\left(t\right))$.

Obviously, $G\left(t\right)=\{M_{f_{t}}\left(G\right);\left[+\right]\}$ for t∈[α,β] forms *dynamically graphic categories*.

With the dynamically colored graph set $M_{f_{t}}\left(G\right)=\left\{G_{i,j}\left(t\right):i\in \left[1,p\right],j\in \left[1,q\right]\right\}$, we have a *dynamically mixed graphic lattice base* as follows:

(33) $$\begin{array}{cc} B(t) & =(G_{1,1}\left(t\right),G_{2,1}\left(t\right),\ldots ,G_{p,1}\left(t\right),\ldots ,G_{s,k}\left(t\right),\ldots ,G_{p,1}\left(t\right),G_{p,2}\left(t\right),\ldots ,G_{p,q}\left(t\right))\\ \; & =(B_{1}\left(t\right),B_{2}\left(t\right),\ldots ,B_{M}\left(t\right)), \end{array}$$

where M=pq. For a graph operation “(•)”, we have a *dynamically mixed graphic lattice*

(34) $$\begin{array}{c} L\left(F_{m,n}\left(t\right)\left(•\right)B\left(t\right)\right)=\left\{H\left(t\right){\left(•\right)}_{k=1}^{M}a_{k}B_{k}\left(t\right):a_{k}\in Z^{0},\;B_{k}\left(t\right)\in B\left(t\right),\;H\left(t\right)\in F_{m,n}\left(t\right)\right\} \end{array}$$

such that each network H(t) is encrypted to another graph $L\left(t\right)=H_{t}{\left(•\right)}_{k=1}^{M}a_{k}B_{k}\left(t\right)$ for t∈[α,β], where $\sum_{k=1}^{M}a_{k}\geq 1$.

**Figure 8:**
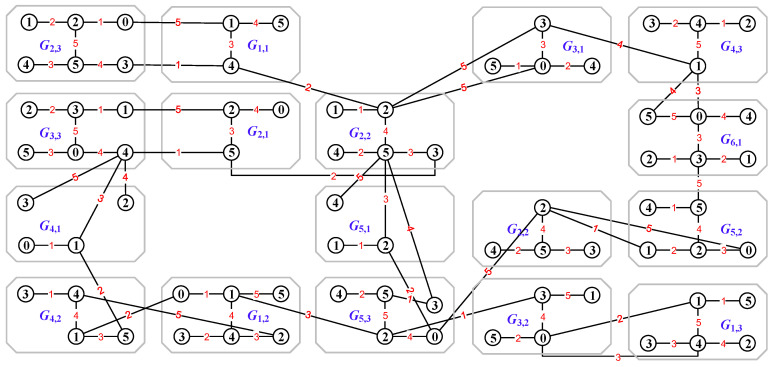
A graph *Q* for illustrating the vertex-coinciding operation in the dynamically mixed graphic lattice shown in Equation (34) based on the every-zero mixed graphic group $M_{f}\left(G\right)=\left\{G_{s,k}:s\in \left[1,6\right],k\in \left[1,5\right]\right\}$ shown in Figure 1.

As the graph operation “(•)” in Equation (34) is the vertex-coinciding operation, an example is shown in Figure 8.

## 5. Summary

To summarize, in the present contribution we firstly defined the graphic category, generalized the mixed graphic groups, and proposed the graphic lattices and various graph-type homomorphisms, from which some useful results were obtained. Based on these results, we then discussed in detail how to encrypt networks in whole by using the mixed graphic groups and the mixed graphic group lattices. In the end, the graphic lattices for the encryption of the dynamic networks were introduced, and the vertex-coinciding operation in the dynamically mixed graphic lattice was illustrated on the basis of the every-zero mixed graphic groups.

## Data Availability

The data used to support the findings of this study are included within the article.
